# Histone Deacetylase Inhibitors, Intrinsic and Extrinsic Apoptotic Pathways, and Epigenetic Alterations of Histone Deacetylases (HDACs) in Hepatocellular Carcinoma

**DOI:** 10.22037/ijpr.2021.115105.15197

**Published:** 2021

**Authors:** Masumeh Sanaei, Fraidoon Kavoosi

**Affiliations:** *Research Center for Non-communicable Diseases, Jahrom University of Medical Sciences, Jahrom, Iran.*

**Keywords:** Neoplasms, HDACS, HDACIs, Apoptosis, Pathway

## Abstract

Epigenetics is the study of heritable modifications in gene expression and reversible forms of gene regulation. Recent *in-vitro *works have indicated that epigenetics plays a significant role in many types of human cancers *e.g.* hepatocellular carcinoma (HCC). Diverse cellular functions are regulated by histone acetylation and deacetylation. Histone deacetylases (HDACs) and histone acetylases (HATs) are enzymes involved in chromatin remodeling histone deacetylation and acetylation respectively. Aberrant protein acetylation, particularly histone deacetylation, has been reported in a broad range of human cancer types. Epigenetic modification by inhibiting HDAC activity is an emerging approach in cancer treatment. HDACIs play their apoptotic roles through multiple mechanisms such as extrinsic/cytoplasmic and intrinsic/mitochondrial molecular mechanisms. Here, we summarize the major classes of HDACs and epigenetic compounds, HDACIs, and also their molecular mechanisms in HCC including intrinsic and extrinsic apoptotic pathways. An online search of different sources including PubMed, ISI, and Scopus was achieved to find suitable data on mechanisms and pathways of HDACs and HDACIs in HCC. The result demonstrated that the dysregulation of HDACs because of histone deacetylation induces HCC. The histone deacetylation can be reversed by HDACIs resulting in apoptosis induction. In conclusion, because histone deacetylation is a potentially reversible change, epigenetic histone modification represents new opportunities for cancer management by reactivation of gene silencing. The inhibition of HDACs by GDACIs can effectively induce apoptosis and suppress cancer cell proliferation. These compounds can engage both intrinsic and extrinsic apoptotic pathways.

## Introduction

Epigenetics is the study of heritable modifications in gene expression and reversible form of gene regulation that is not due to changes in the DNA sequence, it is accepted as ‘‘the assessment and study of changes in gene function that are meiotically and/or mitotically heritable without any structural changes in DNA strand. The epigenetic propose, as proposed by Conrad Waddington in the 1950s, is a branch of cell biology that evaluates the causal interactions between genes and their products, this definition points to all molecular mechanisms modulating the gene expression (1). Epigenetic alterations are crucial for the cell development and differentiation of the different cell types, as well as for normal cellular processes such as the inactivation of X-chromosome in female mammals and gene silencing. These processes can involve chemical modifications include histone acetylation, DNA methylation, ubiquitination, and phosphorylation. Recent *in-vitro *studies have indicated that epigenetics plays a major role in many types of cancers (2-11) such as hepatocellular carcinoma (HCC) (12-16). HCC, the predominant form of adult liver cancer, is one of the most common cancer and remains a major health problem worldwide (17). The dysregulation of the epi-genome and epigenetic control lead to aberrant gene expression, which regulates cancer induction and progression. Therefore, the role of epigenetic compounds has become significant in reverting the malignant phenotype (18, 19). Various cellular actions, comprising tumor suppressor genes (TSGs) expression, cell proliferation, DNA repair, and cell apoptosis, are regulated by acetylation and deacetylation. Histone deacetylases (HDACs) and histone acetylases (HATs) are enzymes involved in chromatin remodeling histone acetylation and deacetylation respectively. These enzymes play an important role in gene expression. HDACs and HATs are used to regulate the gene transcription by removing and/or preserving the acetyl group on speciﬁc histones. This activity can condense or relax the conformation of the DNA, changing the accessibility zones for transcription machinery. These changes result in different cell biological functions, including cell proliferation and diﬀerentiation. Reversible acetylation is one of the most post-translational modifications in eukaryotic cells. Aberrant histone protein acetylation, particularly histone deacetylation, has been shown in numerous human cancer types, dysregulation of HDACs has been implicated in the histo-pathogenesis of cancers (20, 21). Epigenetic modification by inhibiting  HDAC activity is an emerging approach in cancer treatment. Histone deacetylase inhibitors (HDACIs) such as panobinostat, valproic acid (VPA),  vorinostat,  sodium butyrate, trichostatin A, belinostat, and romide-psin have demonstrated antitumor efficacy via activation of various molecular mechanisms. These compounds have strong anti-cancer effects, inducing cell growth arrest, cell differentiation, cell apoptosis (programmed cell death), cell invasion, and suppressing angiogenesis (22, 23). HDACIs play their apoptotic roles through multiple mechanisms such as extrinsic/cytoplasmic and intrinsic/mitochondrial molecular mechanisms (24-26). The present review highlights the role of HDACs in hepatocellular carcinoma (HCC) and the molecular mechanisms through which HDACIs play their apoptotic effects on HCC including extrinsic and intrinsic molecular mechanisms.

**Epigenetic Dysregulation in Cancer induction and Development**

In human cancers, common epigenetic changes include histone modiﬁcation, DNA hypermethylation, and noncoding RNA dysregulation. DNA hypermethylation of CpG islands has emerged as an epigenetic mechanism of silencing *TSGs*. Recent evidence suggest that cancer is associated with the accumulation of cells with aberrant CpG island hypermethylation. Increasing evidence show that DNA hypermethylation of CpG islands is a common molecular mechanism in cancer induction and progression (27). In addition to DNA methylation, numerous molecular mechanisms of histone modification contribute to regulating gene expression epigenetically. Histone acetylation neutralizes the negative charge of DNA strands leads to gene transcription and expression (28). Chromatin remodeling by histone modific-ation is an example of epigenetic gene regulation. Histone deacetylases (HDACs) have recently been shown to modify a variety of other proteins that are involved in diverse cellular processes. The activity of these enzymes results in chromatin compaction, TSGs silencing, and cancer induction (29). Subsequent characterization of TSGs revealed their involvement in cancers and various mechanisms that protect animals against tumorigenesis and tumor development (30).

**Histone acetylases and histone deacetylases biological activity**

**Figure 1:**
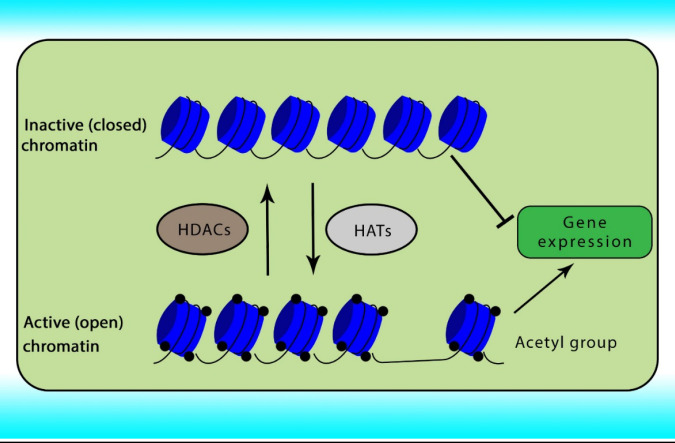
The role of HATs and HDACs on chromatin conformation and gene transcription

Reversible acetylation is one of the most abundant post-translational histone modifications in eukaryotic cells.  Acetylation and deacetylation of histone are achieved by the opposite actions of two groups of enzymes comprising HATs and HDACs. Abnormal expression of HDACs has been indicated in various cancers. The acetylation of histone neutralizes the positive charge of the histone, relaxes the chromatin strands to facilitate the binding of transcription factors, relaxing the DNA strands and enabling greater accessibility of the transcription machinery, and subsequently gene transcription. In contrast, histone deacetylation induces chromatin compaction and gene silencing (31, 32). The role of HATs and HDACs on chromatin conformation has been shown in Figure 1.

**Histone deacetylases classification**

**Figure 2:**
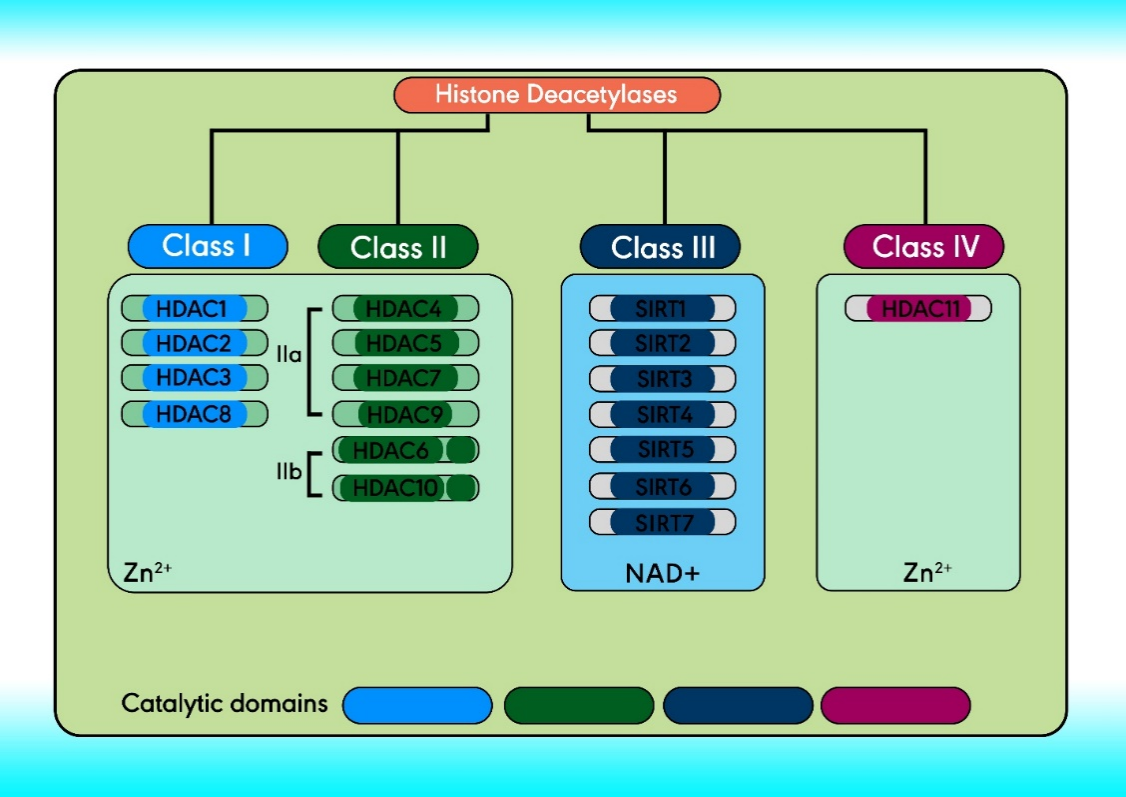
The molecular structure and classification of HDACs

There are 18 HDACs in humans divided into four classes comprising classes I, II, and IV. Based on structure, Class III and the yeast Sir2 protein are homologous, both of which require NAD+ as a cofactor instead of Zn^2+^. Class III HDACs include seven mammalian sirtuins (SIRT1–7), localized in the mitochondria (SIRT3-5), nucleus (SIRT1, and SIRT6-7), and cytoplasm (SIRT2) (33-35). Class I (located within the nucleus) comprises HDACs 1- 3, and 8. Class II can travel between the cytoplasm and the nucleus and can be further subdivided, class IIa and IIb (36). The structure and classification of HDACs are indicated in Figure 2 (37).

**Epigenetic dysregulation in hepatocellular carcinoma**

HCC is characterized by the presence of epigenetic changes, including post-translational modifications of histone and promoter DNA hypermethylation, which affect the expression of numerous genes critical for cancer induction and progression. Strong data suggest that histone deacetylation plays an important role in HCC (38). It is now possible to analyze epigenetic abnormalities associated with various cancers (*e.g.* HCC). Various histone modifications are considered with transcriptional regulatory mechanisms associated with gene expression changes in HCC (39). 

**Histone deacetylases in HCC**

Recent works on TSGs suggest that HDACs are involved in tumor formation and progression of HCC. The expression of HDACs has been linked to clinicopathological factors in HCC. The common HDACs reported in HCC have been shown in Table 1 (40-47). 

**Histone Deacetylase Inhibitors**

**Figure 3:**
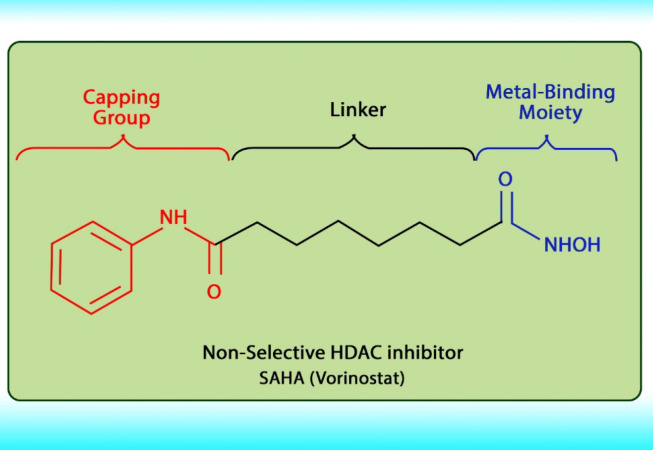
The molecular structure of HDACIs (*e.g*., SAHA).

**Figure 4:**
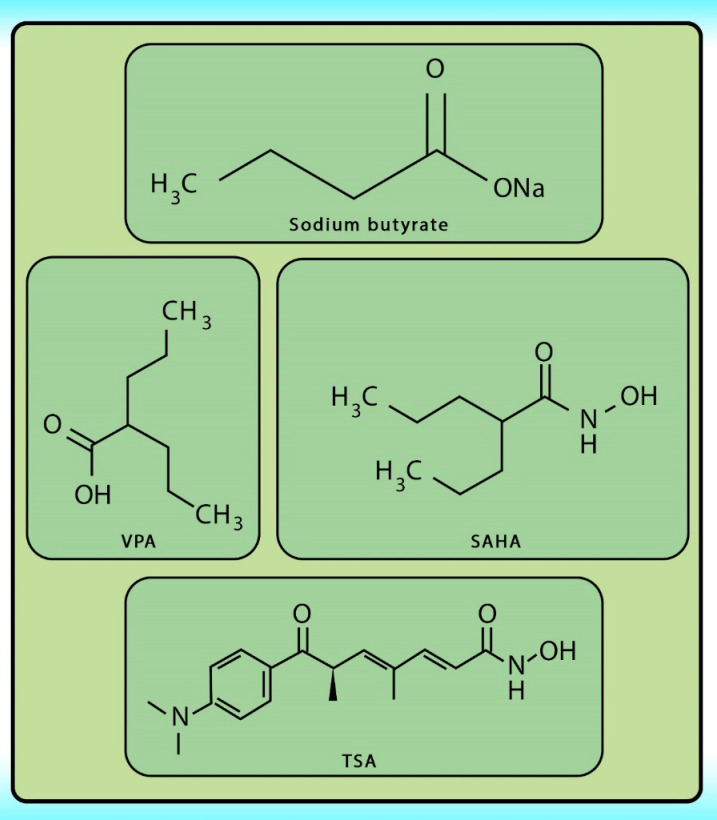
The structures of the most common HDACIs used in HCC

Histone acetylation has been demonstrated to be an important molecular mechanism that controls gene expression. Acetylation and deacetylation of histones cause chromatin decondensation and condensation respectively (48). Steady-state of acetylation results from the balance between the acetylation and deacetylation of histone which determines the level at which a gene is expressed (49). The changes in HAT/HADC activity balance have effect on: (1) alteration in gene expression proﬁle as well as to the change of some signaling pathways; (2) proteasomal degradation; (3) protein kinase C activity and (4) DNA methylation status. In numerous cancers, the shift to an increased acetylation/deacetylation ratio increases chromatin structure relaxation and gene transcription. HDACIs are a diverse group of agents, which vary in biological activity, structure, and specificity. Generally, these compounds contain a capping group, a zinc-binding domain, and a straight-chain linker connecting the two parts (Figure 3). HDACIs act speciﬁcally against HDACs. Based on their chemical structures, they can be classiﬁed into ﬁve classes: (1) short-chain fatty (aliphatic) acids; (2) hydroxamic acids (hydroxamates); (3) cyclic tetrapeptides; (4) benzamides; and (5) sirtuin inhibitors (50). They may also be classified according to their specificity for HDACs. The pan‐deacetylase inhibitors comprising vorinostat, panobinostat, belinostat, trichostatin A, and resminostat. Valproate and butyrate inhibit class I and IIa HDACs, whereas entinostat, mocetinostat, and romidepsin are considered to be the class I specific. Tubacin is HDAC6 specific (51). The HDACIs classification has been indicated in Table 2 (52-54). The chemical structure of common HDACIs used in HCC has been demonstrated in Figure 4.

**Target and molecular mechanisms of HDACIs in HCC**

The level of histone deacetylation is associated with cancer induction and progression. HDACIs play their roles through inhibition of HDACs activity in HCC. The target of HDACIs in HCC has been shown in Table 3 (55-60) and also various molecular mechanisms by which these compounds affect HCC have been indicated in Table 4 (61-67).

**Epigenetic therapy with Histone deacetylase inhibitor**

There are several strategies related to epigenetic therapy in the field of cancer of which is based on the use of HDACIs. These compounds play their roles through various pathways one of which is based on selective induction of apoptosis via the intrinsic pathway and by influencing the expression of proteins such as *Bcl-2, Bcl-XL, Mcl-1,* and *XIAP* (68), as well as through activation of the *DR5, DR4*, and *Fas *(69). Additionally, HDACIs can engage the extrinsic apoptotic pathway through upregulation of DRs expression, reductions in *c-FLIP*, and upregulation of ligands such as TRAIL (70).

**Apoptotic regulators**

Over the last decade, the role of apoptosis regulators such as the *BCL-2* family and their molecular mechanisms has attracted attention. This family can be classified into two groups: a group modulates mitochondrial action and another group regulates the activity of caspases. These proteins regulate the intrinsic apoptotic pathway. Several proteins of the second group, e.g. *XIAP or FLIP* block the activity of caspases. Cross-talk between the extrinsic and intrinsic pathways exists (71). 

Apoptosis is regulated by intrinsic and extrinsic ligands. This process is controlled by the diversity cell signals pathway and is involved in the regulation of cell fate survival or death. As the cross-talk organelles, the mitochondria can connect the different apoptosis pathways (72).

**The apoptotic pathways**

There are two major molecular mechanisms of cell death including necrosis and apoptosis. The cells undergo necrosis because of external injury, while apoptosis is a programmed cell death because of internal or external stimuli which are controlled by numbers of complex proteins. It occurs through two main pathways comprising extrinsic and intrinsic apoptotic pathways. 

**Figure 5:**
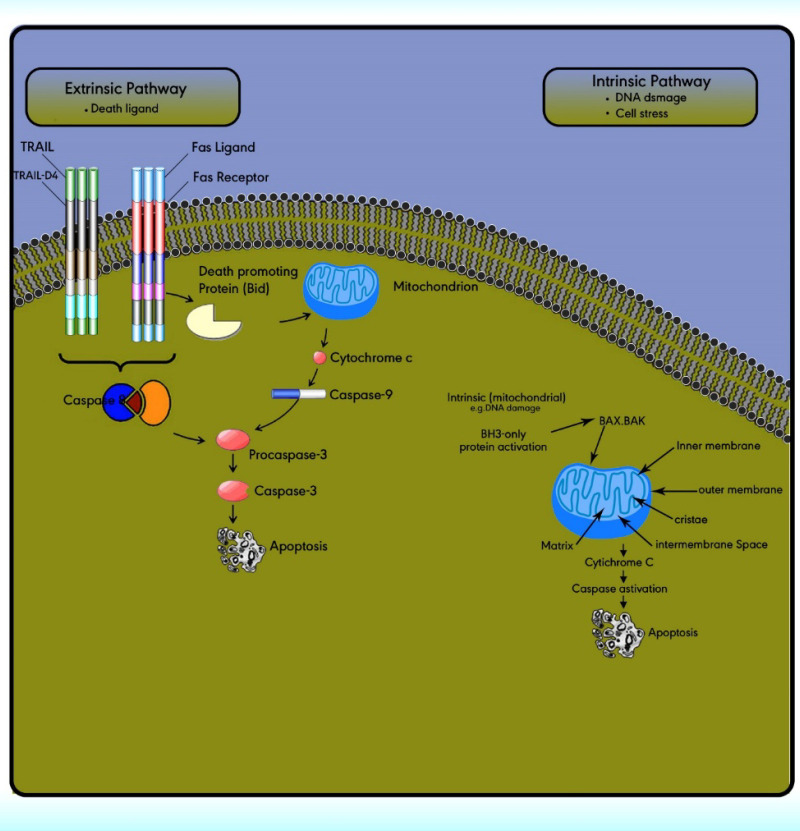
Apoptotic pathways, intrinsic and extrinsic. The extrinsic pathway is triggered through the death ligand. While the intrinsic pathway is triggered by cell stress or DNA damage. Both pathways lead to a final common pathway which activate caspases that cleave regulatory molecules, lead up to the death of the cell

The extrinsic (death receptor) apoptotic pathway is triggered through the Fas death receptor (73). The intrinsic (mitochondrial) apoptotic pathway results in the release of cytochrome‐c from the mitochondria which activates the death signal (74). Both pathways lead to a final common pathway which activate caspases that cleave regulatory molecules, lead up to the death of the cell (Figure 5) (75).

**Extrinsic pathway or death receptor pathway**

The extrinsic apoptotic pathway includes numerous protein members comprising the death receptors (DRs), the Fas ligand, the Fas‐associated death domain, the Fas complexes, caspases 8, and 10 (Figure 5). The extrinsic pathway is initiated with the ligation of cell surface receptors. DRs may belong to the tumor necrosis factor family (TNF), which is composed of several members. *Fas* is a TNF and is also called *Apo‐1 or CD95*. Currently, six DRs are known, comprising TNF receptor 1, Fas, p55, p60, DR3, TNF-related apoptosis-inducing ligand receptor 1, TRAIL-R2, and DR6 (76). 

The extrinsic/death receptor pathway is initiated via binding of the cell surface death receptors, DRs, with their ligands. After activation, the intracellular domains of these receptors bind to the adaptor protein Fas-associated death domain (FADD) or TNFR1-associated death domain protein (TRADD) to form the death-inducing complex with the recruitment of pro-caspase 8. Then, the activated procaspase 8 serves as the ‘initiator’ caspase, further activating downstream effectors such as caspase 7 to initiate apoptosis induction (77). In some cell types, the activation of caspase 8 may be the only requirement to execute death (78).

**Intrinsic pathway or the mitochondrial pathway**

The intrinsic pathway is activated via the loss of cell growth factor signals or in response to intracellular stimuli. Mitochondria have an outer membrane (OM), an inner membrane (IM), and an intermembrane space (IMS). The IMS includes several proteins involved in cell apoptosis and cell death induction, such as cytochrome c, Smac/DIABLO, Omi/HtrA2, apoptosis-inducing factors, and EndoG.

**Figure 6:**
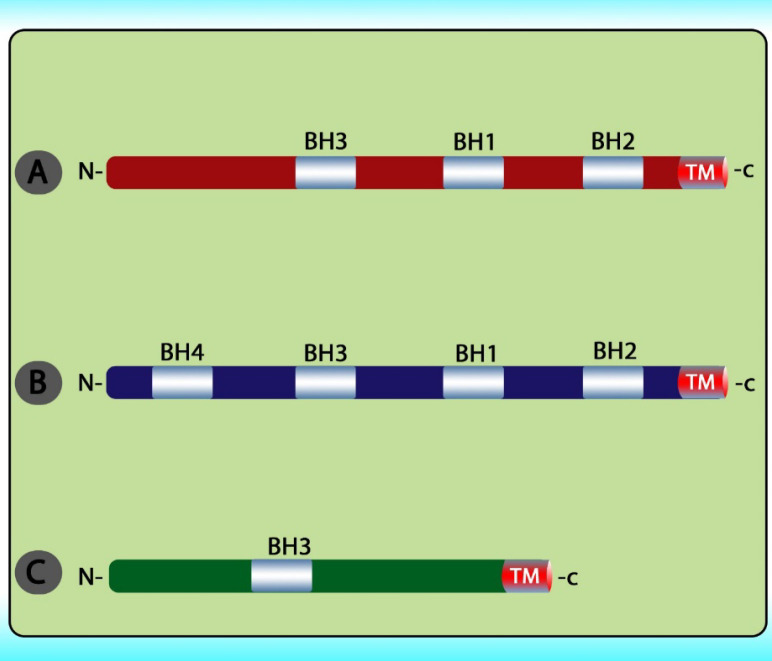
Overview of the BCL2 protein family. This family is divided into three groups including (A) multi-domain pro‑apoptotic BCL2 family proteins; (B) multi‑domain anti‑apoptotic BCL2 family proteins; and (C) BH3‑only pro‑apoptotic BCL2 family proteins

 The stimuli change the permeability of the inner mitochondrial membrane and release the pro-apoptotic proteins from the intermembrane space into the cytoplasm. After the release of these proteins into the cytoplasm, cytochrome c stimulates apoptosome formation followed by caspase 9 activation. Caspase 9 activates caspases 3, 6, and 7 leads to cell death (Figure 5) (79). The intrinsic pathway is controlled through interactions between the members of the Bcl-2 protein family. This family contains 20 proteins divided into 3 groups: one anti-apoptotic group and two pro-apoptotic groups. The members of this family can be identified via *Bcl-2* homology domains (BH1 to BH4). Each of the BH domains has a different action. Whereas most anti-apoptotic proteins comprise all domains and protect cells exposed to various conditions (Figure 6). These proteins are divided into two subgroups. The first group contains BH3-only domain proteins whereas the second group contains the BH 1-3 domains. Anti-apoptotic proteins exert their function by binding the member’s *Bak and Bax*, preventing mitochondrial damage (80). The function and classification of the *BCL-2* family have been shown in Table 5 (81-88).

**Table 1:** The common HDACs reported in HCC

**Class**	**Member**	**Localization**
class I	HDAC 1-3, and 8	Nucleus
IIa	HDAC4-7, and 9	Nucleus/cytoplasm
IIb	HDAC6 and 10	Cytoplasm
IV	HDAC 11	Nucleus

**Table 2:** HDACIs classification

**Class**	**Member**	**Target (HDAC specifity)**
Short-chain fatty acids	Valproic acid, Sodium butyrate	I, IIa I, II
Hydroxamic acids	TSA SAHA CHR-3996 Belinostat Givinostat Panobinostat Resminostat Quisinostat Abexinostat Rocilinostat Practinostat Tubacin	Pan Pan I Pan Pan Pan Pan Pan Pan II I, II, IV IIb
Cyclic tetrapeptides	Romidepsin Apicidin	I I
Benzamides	4SC202 MS-275 Mocetinostat MGCD0103 Entinostat Tacedinaline CI-994	I I I, IV I I I I
Sirtuins inhibitors	EX-527 Cambinol Sirtinol Nicotinamide	SIRT 1 SIRT 1, and 2 SIRT 1, and 2 3 (all classes)

**Table 3:** The target of HDACIs in HCC

**Class**	**Member**	**Target HDAC class**	**Cell line**	**Reference**
short-chain fatty acids	Valproic acid (VPA),	class I, IIa	hepatocellular carcinoma HepG 2 cells	A
Aliphatic fatty acid	sodium butyrate	class I, IIa	SMMC-7721 and HepG2 cells	B
Aliphatic fatty acid	sodium butyrate	class I, IIa	HCC SMMC-7721 and HepG2 cells	C
Hydroxamic acid	TSA	Pan	HCC SMMC-7721 and HepG2 cells	C
Hydroxamic acid	trichostatinA (TSA)	Pan	Hep3B,HepG2, Hep3B	D
Hydroxamates	(SAHA)	Pan	Bel-7402, HepG2	E, F

**Table 4:** HDACIs pathways in HCC

**Class**	**Member**	**Apoptosis pathway**	**Tissue or cell line**	**Reference**
short-chain fatty acids	Valproic acid (VPA),	Intrinsic	HepG2, BEL-7402, and SMMC-7721	A
Aliphatic fatty acid	sodium butyrate	Intrinsic And Extrinsic	HuH-6, HepG2, HuH-6, HuH-7, Hep-G2, and PLC/PRF/5 cells	B, C
Hydroxamic acid	TrichostatinA (TSA)	Intrinsic	HepG2, MH1C1, Hepa1–6 and Hep1B	D
Hydroxamates	Suberoylanilide hydroxamic acid (SAHA)	Intrinsic	HCCLM3, 7703K, SMMC7721, BEL7402, and HepG2 cell lines	E, F, G

**Table 5:** The function and classification of the *BCL-2* family

**Group**	**Member**	**Mechanism of action**	**Subcellular localization**	**Domain**
Anti-apoptotic BCL-2 proteins	BCL-2 (B-cell lymphoma protein 2)	It inhibits apoptosis by the preservation of mitochondrial membrane integrity.	(1) Nuclear envelope (2) Outer mitochondrial membrane (3) Membrane of the endoplasmic reticulum membrane (ER)	Contain four BH (1-4) domains
	BCL-W (Bcl2-like protein 2)	It reduces cell apoptosis under cytotoxic conditions.	Exclusively on the mitochondrion	
	BCL-XL (BCL-extra long)	It inhibits cytochrome c release that inhibits activation of the cytoplasmic caspase cascade by cytochrome c	The transmembrane molecule in the mitochondria	
	MCL1 (myeloid cell leukemia-1)	It interacts with Noxa, BAK1, BCL2L11, Bcl-2-associated death promoter, PCNA.	Nucleus, mitochondria	
pro-apoptotic proteins	BAK (BCL-2-antagonist/killer-1)	It undergoes conformational changes to form larger aggregates during apoptosis	Integral mitochondrial membrane protein	Contain three conserved BH domains
	BAX (BCL-2-associated×protein)	Release of apoptogenic factors like cytochrome c, activation of caspase cascade	Cytosol	
	BOK (BCL-2 related ovarian killer)	Major functions of BOK are exerted on the ER membranes and the Golgi and that it induces apoptosis in a manner dependent on BAK and BAX.	Nucleus	
pro-apoptotic BH3-only proteins	BID (BH3 interacting domain death agonist)	Bid, free ‘activator’ type BH3 only protein, which can then activate Bak and Bax.	Mitochondria	BH3-only proteins that have homology to the BCL-2 family proteins in only a single domain, the BH3 domain
	BIM Bim (Bcl-2 interacting mediator of cell death)	It promotes cell death.	The mitochondrial outer membrane (MOM)	
	BAD (BCL-2 associated death promoter)	Dephosphorylated BAD forms a heterodimer with Bcl-xL, and Bcl-2, and Bcl-xL, inactivating them and thus allowing Bax/Bak-triggered apoptosis	Free in mitochondria	
	BIK (BCL-2 interacting killer)	It promotes Ca2 release from, activation of ER-localized Bax/ Bak.	ER	
	BMF BCL-2 modifying factor (BMF)	It acts as an initiator of the intrinsic apoptosis pathway.	MOM	
	HRK (Hara-kiri)	Hrk is a critical downstream effector of the JNK dependent mitochondrial apoptotic signaling pathway.	MOM	
	NOXA (Latin for “damage”)	A selective inhibitor of MCL1	MOM	
	PUMA (p53 upregulated modulator of apoptosis)	It promotes ER Ca2 pool depletion during thapsigargin-induced apoptosis with a Bax-dependent mechanism.	MOM	

## Conclusion

Histone acetylation and deacetylation are important mechanisms to regulate gene expression. The level of HDACs is generally dysregulated in HCC, it is well accepted that epigenetic events, such as histone modification play an important role in normal biological processes and tumorigenesis and that the epigenetic status is altered during cancer initiation. Because histone deacetylation is a potentially reversible change, the epigenetic histone modification represents new opportunities for cancer management by reactivation of gene silencing. The inhibition of HDACs by GDACIs can effectively induce apoptosis and suppress cancer cell proliferation. These compounds can engage both intrinsic and extrinsic apoptotic pathways. 
